# “Leader–Follower” Dynamic Perturbation Manipulates Multi-Item Working Memory in Humans

**DOI:** 10.1523/ENEURO.0472-22.2023

**Published:** 2023-11-20

**Authors:** Qiaoli Huang, Minghao Luo, Yuanyuan Mi, Huan Luo

**Affiliations:** 1 School of Psychological and Cognitive Sciences, Peking University, Beijing 100871, China; 2 PKU-IDG/McGovern Institute for Brain Research, Peking University, Beijing 100871, China; 3 Beijing Key Laboratory of Behavior and Mental Health, Peking University, Beijing 100871, China; 4 Department of Psychology, Max Planck Institute for Human Cognitive and Brain Sciences, Leipzig 04103, Germany; 5 Department of Psychology, School of Social Sciences, Tsinghua University, Beijing 100084, China

**Keywords:** dynamic perturbation, memory manipulation, short-term plasticity, time-based, working memory

## Abstract

Manipulating working memory (WM) is a central yet challenging notion. Previous studies suggest that WM items with varied memory strengths reactivate at different latencies, supporting a time-based mechanism. Motivated by this view, here we developed a purely bottom-up “Leader–Follower” behavioral approach to manipulate WM in humans. Specifically, task-irrelevant flickering color disks that are bound to each of the memorized items are presented during the delay period, and the ongoing luminance sequences of the color disks follow a Leader–Follower relationship, that is, a hundreds of milliseconds temporal lag. We show that this dynamic behavioral approach leads to better memory performance for the item associated with the temporally advanced luminance sequence (Leader) than the item with the temporally lagged luminance sequence (Follower), yet with limited effectiveness. Together, our findings constitute evidence for the essential role of temporal dynamics in WM operation and offer a promising, noninvasive WM manipulation approach.

## Significance Statement

WM is known to be the sketch pad of conscious thought that allows us to temporally hold and manipulate limited amounts of information to guide future behavior. A major challenge in the WM field concerns how multiple items could be simultaneously retained while not being confused with each other. Previous work advocates a time-based mechanism, with the item with stronger strength firing at earlier latency than that with weaker memory. Motivated by the time-based view, here we developed a novel behavioral approach, namely the Leader–Follower dynamic perturbation, to alter WM performance in humans. Our findings constitute new evidence for a time-based WM mechanism and offers a brand new behavioral approach to directly manipulate WM but with the need for replication.

## Introduction

Manipulating working memory (WM) is an important yet challenging notion and would also provide crucial causal evidence for the WM neural mechanism. It is suggested that WM information undergoes reactivation or refreshing to overcome memory decay during the delay period (Curtis and D’Esposito, 2003; Vogel and Machizawa, 2004), a process that facilitates memory storage via short-term neural plasticity (STP) principles (Wang et al., 2006; Mongillo et al., 2008; Miller et al., 2018). When multiple items are retained, previous models suggest that the item-specific reactivations compete with each other over time (Oberauer and Lewandowsky, 2008, 2011), wherein an individual item fires at varied phases according to its respective memory strength (Lisman and Idiart, 1995; Lisman and Jensen, 2013). The item with stronger memory strength, given its higher neural excitability, fires at an earlier latency, whereas the less excitable item reactivates relatively late (Siegel et al., 2009; Bahramisharif et al., 2018; Huang et al., 2018, 2021), enabling the transformation of memory strengths into neural activities with varied latencies. Hence, a potential yet unexplored WM manipulation approach is to alter the temporal relationship between item-specific reactivations during retention so their relative memory performance could be modified.

Previous research on noninvasive WM modulation in humans has highlighted several approaches, such as frequency-specific transcranial magnetic stimulation (TMS) and transcranial alternating current stimulation (tACS; Sauseng et al., 2009; Hoy et al., 2015; Beynel et al., 2019). Moreover, presentation of a retro cue could prioritize recalling performance via top-down attentional modulations (Griffin and Nobre, 2003; Landman et al., 2003; Oberauer and Hein, 2012; Myers et al., 2017). Recently we developed a purely bottom-up behavioral dynamic perturbation approach to interfere with the multi-item neural dynamics of sequence WM (Li et al., 2021). Notably, this approach draws on many theoretical models and empirical findings. First, color features, even task irrelevant, tend to be automatically bound to memorized items, that is, object-based WM (Luck and Vogel, 1997; Johnson et al., 2008; Huang et al., 2018; Li et al., 2021). Accordingly, presentation of color disks that are attached to memorized items could possibly reactivate and even modify memories. Second, although it has been suggested that WM information is stored in an active or activity-silent manner (Goldman-Rakic, 1995; Curtis and D’Esposito, 2003; Rose et al., 2016; Wolff et al., 2017; Miller et al., 2018), memory manipulation still relies on active states to drive STP-based modifications of synaptic efficacies (Masse et al., 2019, 2020; Barbosa et al., 2020). This idea is akin to the reconsolidation process in long-term emotional memories, whereby the stored information is rendered labile after being retrieved so that new information could be incorporated into and modify old memories (Schiller et al., 2010; Agren et al., 2012; Lane et al., 2015). Finally, flickering color disks have been found to be able to tag item-specific neural reactivations (Huang et al., 2018). Therefore, altering the temporal relationship between luminance sequences of color disks that are linked to each memorized item would presumably perturb the multi-item reactivation profiles to manipulate their memory performances. These points motivate the dynamic perturbation approach developed in our previous study, wherein we demonstrate that temporally synchronized luminance sequences disrupt the recency effect, whereas temporally independent luminance sequences keep the recency intact (Li et al., 2021). Nevertheless, the recency effect is just a behavioral index for the WM sequence, and an efficient bottom-up behavioral approach to modulate multi-item WM performance at a general level is still lacking.

Here, we developed a new “Leader–Follower” approach for WM manipulation during which participants temporarily hold two or three items simultaneously. We introduced a temporal lag at hundreds of milliseconds based on previous findings (Lisman and Idiart, 1995; Mongillo et al., 2008; Mi et al., 2017; Bahramisharif et al., 2018; Huang et al., 2018; Herweg et al., 2020) to the luminance sequences of flickering color disks during retention. Specifically, one luminance sequence (Leader), although a randomly generated white noise that does not contain any regularities, always precedes another sequence (Follower) by a certain temporal lag. We hypothesize that the item bound to the Leader luminance sequence reactivates earlier than the item with the Follower sequence and therefore has better memory performance. Four behavioral experiments on 120 participants provided modest evidence that the item associated with the temporally advanced luminance sequence turns out to have better memory performance than the item modulated by temporally lagged luminance sequence. Together, our results not only offer a new bottom-up behavioral approach to manipulating WM performance but also constitute new evidence supporting the critical role of temporally sequenced reactivations in multi-item WM.

## Materials and Methods

### Participants

One hundred thirty-one participants (50 males, age ranging from 17 to 25 years) took part in five experiments. Two participants in experiment 1, two in experiment 2, three in experiment 3, and four in experiment 4 were removed because of their extreme memory performance (beyond 2.5 * σ) or for not finishing the whole experiment, which resulted in 30 participants for each experiment. An a priori power analysis run in G*Power software (Faul et al., 2009) revealed that to obtain an effect of Cohen’s *d* = 0.55 for a two-sided paired sample *t* test with a power of 0.8, data from 28 participants needed to be collected. The expected effect size of interest for a difference in normalized target probability between the Leader and the Follower condition was derived based on a pretest on 25 subjects, using a similar paradigm as in experiment 1. All the participants had normal or corrected-to-normal vision with no history of neurologic disorders. They were naive to the purpose of the experiments and provided written informed consent before the start of the experiment. All experiments were conducted in accordance with the Declaration of Helsinki and have been approved by the Research Ethics Committee at Peking University.

### Stimuli and tasks

Participants sat in a dark room in front of a Display++ monitor with a 100 Hz refresh rate and a resolution of 1920 * 1080 with their head stabilized on a chin rest. Participants performed a multi-item working memory task. At the beginning of the trial, multiple bars (0.56° × 1.67° visual angle; two bars in experiments 1 and 2, three bars in experiments 3 and 4) were simultaneously presented at different locations on the screen in different colors. Participants were instructed to memorize the orientations of the bars and their colors (experiments 1 and 3) or their spatial locations (experiments 2 and 4). During memory maintenance, colors disks flickered for 5 s, and participants performed a central fixation task by monitoring an abrupt luminance change of the central fixation cross. Finally, participants needed to rotate a horizontal test bar by pressing corresponding keys to one instructed memorized orientation as precisely as possible, without a time limit. The luminance of the flickering disk was randomly generated (ranging from 0 cd/m^2^ to 15 cd/m^2^) and was tailored to have equal power at all frequencies by normalizing the amplitudes of its Fourier components before applying an inverse Fourier transform separately for the red and blue colors. The colors and the spatial locations of the bars and disks were carefully balanced across trials to eliminate possible color-specific or spatial-specific effect. Participants completed 192 trials in total in experiments 1 and 2, which took ∼1 h, and they completed 162 trials in total in experiments 3 and 4, which also took ∼1 h.

### Experiment 1

In each trial, after a 0.5 s fixation period, two bars in red and blue were presented at a 3° visual angle above and below the fixation for 2 s. The orientations of the two bars were chosen randomly, with a difference of at least 10°. The colors and spatial locations of the two bars were counterbalanced across trials. Participants were instructed to memorize the orientations and colors of the bars. After a blank interval (0.6 ∼ 1 s), two disks (3° in radius) with the same colors as the two memorized bars were presented at the left or right side of the fixation (7° in eccentricity) for 5 s. The colors and spatial locations of the two disks were counterbalanced across trials. Crucially, the luminance of the two color disks was continuously modulated according to two 5 s temporal sequences ranging from dark (0 cd/m^2^) to bright (15 cd/m^2^). Specifically, in each trial, a 5 s temporal sequence was first randomly generated (Leader sequence), and then we shifted the Leader sequence 200 ms rightward and moved the final 200 ms segment of the Leader sequence to the beginning to generate a new sequence (Follower sequence). Note that the luminance sequences were generated anew in each trial, and it was quite hard to differentiate between the Leader and Follower sequences. Throughout the 5 s maintenance period, participants performed a central fixation task by continuously monitoring an abrupt luminance change of the central fixation cross while simultaneously holding the two bars. The fixation task is used to eliminate the effect of attentional bias. After finishing the fixation task, a horizontal test bar in red or blue was presented to instruct participants to recall the orientation of the red or blue bar and rotate the test bar to the target orientation as precisely as possible.

### Experiment 2

Experiment 2 had the same stimuli and similar paradigm as experiment 1. The only difference was that instead of requiring participants to memorize the orientations of two bars and their colors, we asked participants to memorize the orientations and spatial locations of two bars. Specifically, after finishing the fixation task, a retrospective cue (top or bottom character) was presented for 1 s to instruct participants to recall the orientation at the top or bottom. Then a horizontal bar in white was presented, and participants rotated it to the instructed memorized orientation. Therefore, in experiment 2, color information was totally task irrelevant.

### Experiment 3

Experiment 3 was a three-item memory task with one task similar to that in experiment 1. In each trial, three bars colored in red, blue, and green were presented at the same eccentricity as the fixation (3° visual angle) for 3 s. The orientations of the three bars were chosen randomly, with a difference of at least 10° between any two orientations. The colors and spatial locations of the three bars were randomized. Participants were instructed to memorize the orientations and colors of the bars. After a blank interval (0.6 ∼ 1 s), three disks (3° in radius) with the same colors as the three memorized bars were presented at a 7° eccentricity to the fixation for 5 s. The disk and bar with the same color were presented in the same direction of the fixation but different spatial locations. Similarly, the luminance of the three color disks was continuously modulated according to three 5 s temporal sequences ranging from dark (0 cd/m^2^) to bright (15 cd/m^2^). Specifically, in each trial, a 5 s temporal sequence was first randomly generated (Leader sequence), and then we shifted it 150 ms rightward to generate the Follower_1st_ sequence. Similarly, we shifted the Follower_1st_ sequence 150 ms rightward to generate the Follower_2nd_ sequence. Although the three sequences were presented simultaneously, their temporal relationship showed that Leader lead Follower_1st_ 150 ms, Follower_1st_ lead Follower_2nd_ 150 ms, and Leader lead Follower_2nd_ 300 ms. After finishing the fixation task, a horizontal bar in red, blue, or green was presented, and participants were instructed to recall the orientation of the red, blue, or green bar and rotate it to the target orientation as precisely as possible. 

### Experiment 4

Experiment 4 had the same stimuli and a similar paradigm as experiment 3, except that instead of requiring participants to memorize the orientations and colors of the three bars, we asked them to memorize the orientations and spatial locations of the three bars. Specifically, after finishing the fixation task, a retrospective cue (left, middle, or right character) was presented for 1 s to instruct participants to recall the orientation at the left, middle, or right location (horizontal direction). Then, a horizontal bar in white was presented, and participants were asked to rotate it to the instructed memory orientation. Therefore, as experiment 2, the color information was also totally task irrelevant in experiment 4.

### Data analysis

To quantify the memory performance for each item, a probabilistic mixture model (Bays et al., 2009) was applied to fit behavioral performance. Specifically, the mixture model simultaneously characterizes the contribution of the memory for the target item, nontarget item, and random guess to the final report. Specifically, this model calculates the probability of correctly reporting the feature value of the target item with some variability, the probability of mistakenly reporting the feature value of one of the other nontarget items held in memory with the same variability, and the probability of generating a random response unrelated to either target or nontarget items. In the present study, we focused on target probability because it represents the memory accuracy for the target and has been widely used to quantify memory performance (Gorgoraptis et al., 2011; Van Ede et al., 2018; Li et al., 2021). Moreover, considering that the target probability is not normally distributed, we performed an empirical logit transformation as follows: logit(p) = l n((*p* + 1/2 n) / (1 − *p* + 1/2 n)), where *p* is the target probability and *n* is the number of observation transformations (De Smith, 2018). The normalized target probabilities were used for further statistical tests in all the experiments. In addition, memory precision was estimated by calculating the reciprocal of the circular SD of response error (the circular difference between the reported orientation and the true target orientation).

### Statistics

Classical frequentist statistics, for example, repeated ANOVA and paired *t* tests, were applied to test experimental effect. Considering there are three conditions in experiments 3 and 4, a Holm correction was applied for *post hoc* analysis.

Apart from classical frequentist statistics, we also implemented Bayesian statistics using JASP software (version 0.16.4.0). Specifically, for the paired *t* test, we provided Bayes factor BF_10_, which quantifies how many times the observed data are more likely under the alternative hypothesis that postulates the presence of the experimental effect (e.g., the perturbation effect) than under the null hypothesis. For repeated ANOVA, we reported the inclusion Bayes Factor, BF_incl_, which reflects the evidence for all models with a particular experimental effect compared with all models without that particular effect. A Bayes factor >1 can be interpreted as evidence against the null; one convention is that a Bayes factor >3 can be considered as substantial evidence against the null and vice versa (a Bayes factor smaller than one-third indicates substantial evidence in favor of the null model; Wetzels et al., 2011). Bayesian *post hoc* tests were applied in experiments 3 and 4. We reported the uncorrected Bayes factor, BF_10,U_, and posterior odds, which have been corrected for multiple testing by fixing the prior probability that the null hypothesis holds across all comparisons to 0.5 (Westfall et al., 1997). 

### Data availability

Data and the associated code are available from the Open Science Framework at https://osf.io/cpvdk/.

## Results 

### Leader–Follower dynamic perturbation modulates two-item memory performance (experiment 1)

**Figure 1.**
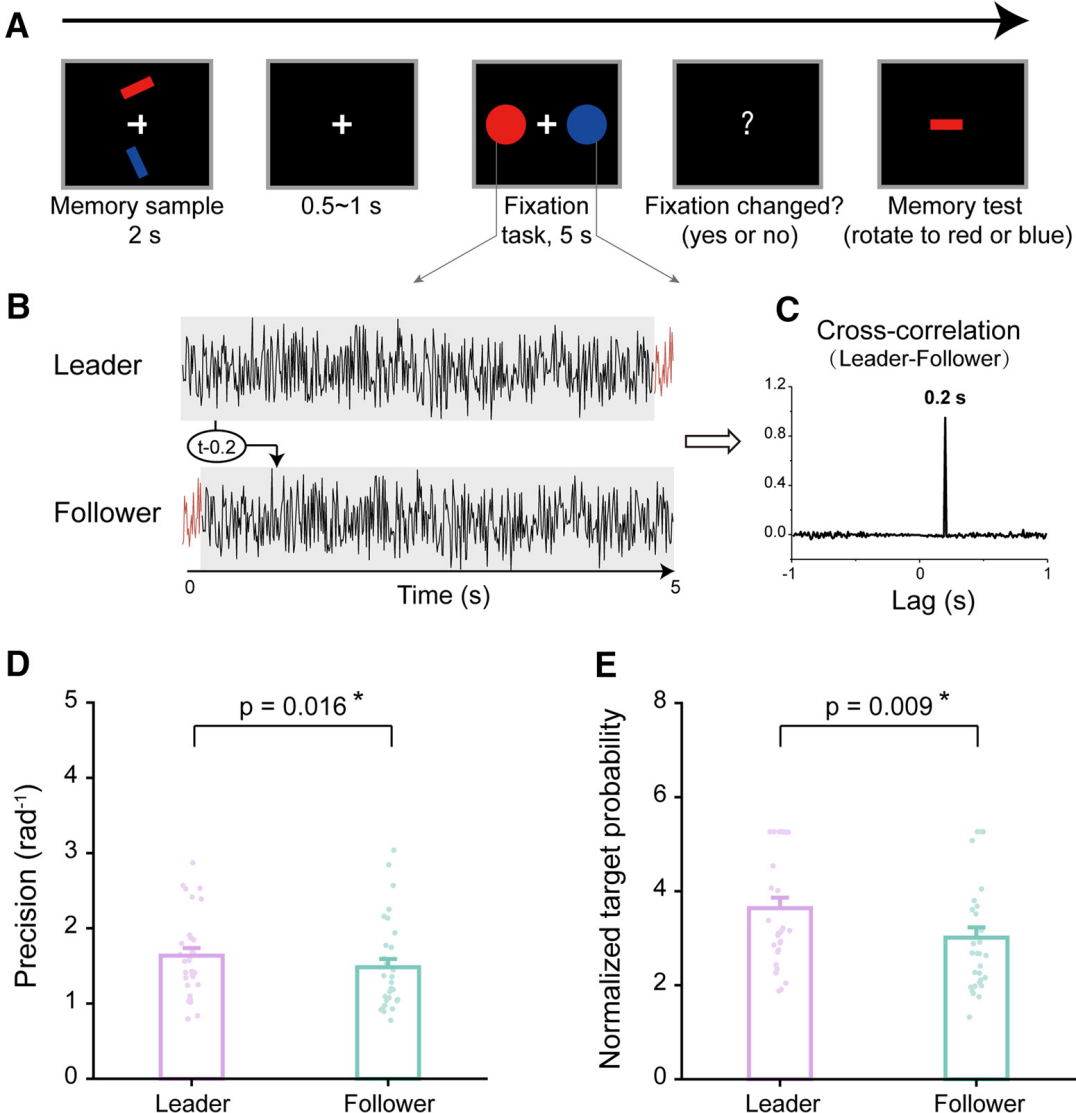
Leader–Follower dynamic perturbation during retention modulates two-item memory performances (experiment 1, *N* = 30). ***A***, Leader–Follower dynamic perturbation paradigm. In each trial, participants were presented with two bars, and they memorized their orientations and colors. During the memory test, participants adjusted the orientation of a probe bar to match that of the memorized bar that was the same color as the probe. During the 5 s delay period, participants performed a central fixation task while two task-irrelevant flickering disks of the same color as each of the memorized bars (blue and red) were presented bilaterally, with their luminance continuously modulated by two 5 s temporal sequences, Leader or Follower sequences, respectively. The color, spatial location, and Leader–Follower conditions were counterbalanced across trials. ***B***, The Leader temporal sequence was a 5 s white noise randomly generated per trial, and the Follower sequence was created by circular shifting the Leader sequence 200 ms rightward. Note that the two sequences were presented simultaneously rather than asynchronously. ***C***, The Leader–Follower cross-correlation over time as a function of temporal lag, peaking at 200 ms. ***D***, Memory performance. Grand averaged (mean + SEM) memory precision during recalling test for Leader (purple) and Follower (turquoise) conditions, with dots denoting individual participants. ***E***, Same as ***D***, but for normalized target probability; *p < 0.05. Extended Data Figure 1-1 shows target probability without normalization. Extended Data Figure 1-2 shows additional parameter results (nontarget and random guess probability).

Thirty participants performed a two-item memory task in experiment 1 (Fig. 1*A*). In each trial, two bars were simultaneously presented at the top and bottom locations, and participants needed to memorize both orientations and colors of the two bars over a 5 s delay period while performing a central fixation task. During the recalling phase, participants adjusted the orientation of a probe bar to match that of the memorized bar of the same color as the probe. Crucially, during the 5 s delay period, two task-irrelevant disks with the same colors as one of the memorized bars, one red and one blue, were bilaterally presented, and their luminance was continuously changing according to two 5 s temporal sequences (Fig. 1*B*). The two luminance sequences were designed to have a specific temporal relationship, with their cross-correlation coefficient peaking at a 200 ms lag (Fig. 1*C*). Specifically, one sequence randomly generated per trial (Leader sequence) would be used to generate the other by introducing a 200 ms lag (Follower sequence). In other words, to generate two random sequences with a fixed time lag, we temporally shifted one sequence (Leader) rightward by 200 ms to generate the Follower sequence. Moreover, to ensure their simultaneous occurrence, we cut the last 200 ms segment of the Follower sequence and shift it to its beginning so that the Leader and Follower sequences still had a fixed circular temporal lag. Finally, the color, spatial location, and Leader–Follower conditions were counterbalanced across trials.

10.1523/ENEURO.0472-22.2023.f1-1 Figure 1-1 ***A***, Target probability for the Leader (purple) and Follower (turquoise) conditions, with dots denoting individual subjects in experiment 1. ***B–D***, Same as ***A***, but for experiments 2–4. Correction for multiple comparisons was applied to experiments 3 and 4. Download Figure 1-1, TIF file.

**Figure 2.**
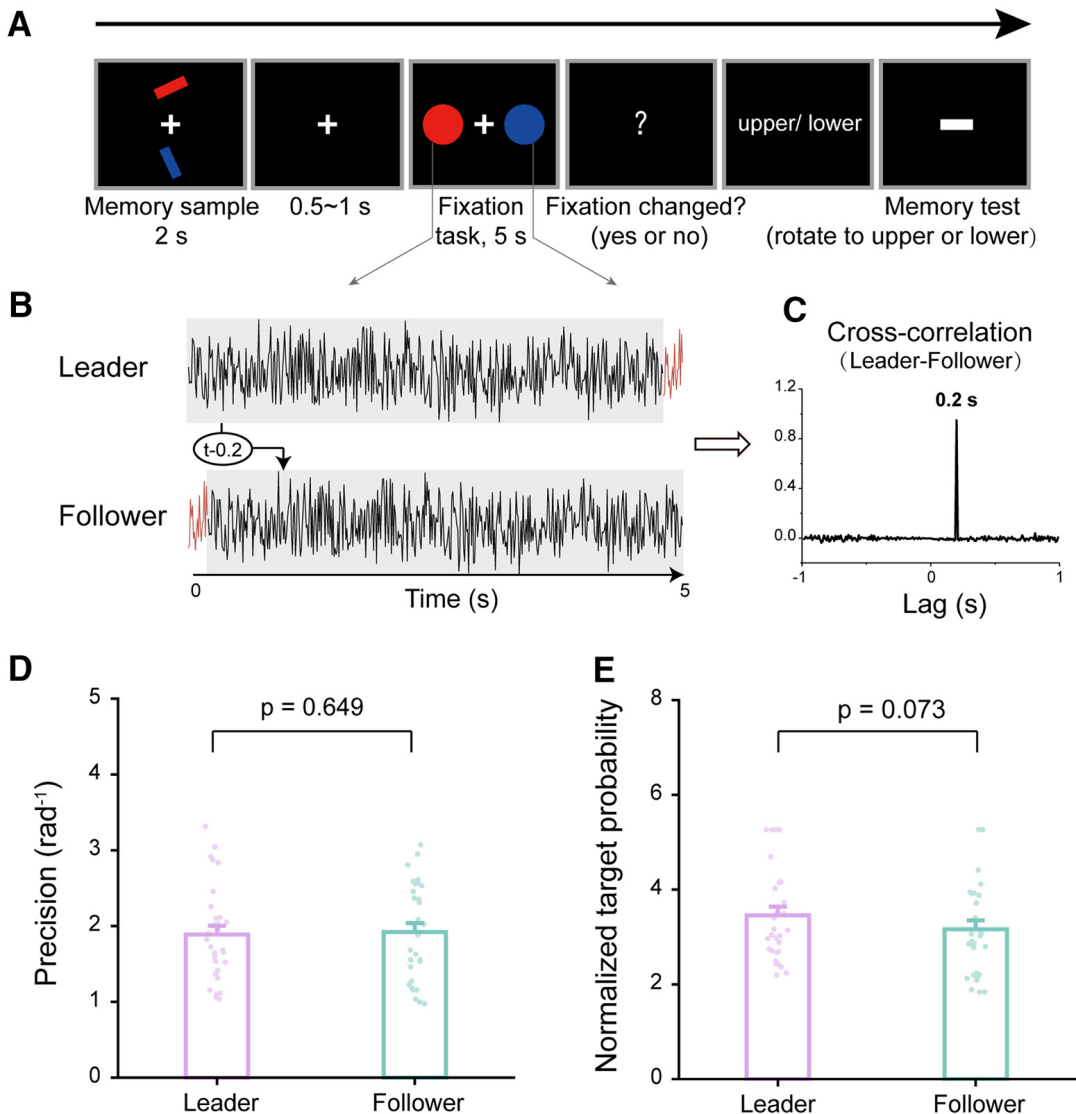
Task-irrelevant Leader–Follower dynamic perturbation (experiment 2, *N* = 30). ***A***, Task-irrelevant dynamic perturbation paradigm. Experiment 2 was the same as experiment 1, except that participants needed to memorize the orientations and locations (top or bottom) of the two bar stimuli regardless of their colors. During the memory test period, a location cue (top or bottom) was first presented, and based on this participants rotated the horizontal white bar to the corresponding memorized orientation. Critically, a Leader–Follower dynamic perturbation as in experiment 1 was applied during the delay period; that is, two disks of the same color as each of the memorized bars (blue and red) were presented bilaterally, with their luminance continuously modulated by a Leader or Follower sequence, respectively. ***B***, The Leader temporal sequence was a 5 s white noise randomly generated per trial, and the Follower sequence was created by circular shifting the Leader sequence 200 ms rightward. The two luminance sequences were presented simultaneously rather than asynchronously. ***C***, The Leader–Follower cross-correlation over time as a function of temporal lag, peaking at 200 ms. ***D***, Memory performance. Grand averaged (mean + SEM) memory precision during recalling test for Leader (purple) and Follower (turquoise) conditions, with dots denoting individual participants. ***E***, Same as ***D***, but for normalized target probability. Extended Data Figure 1-1 shows target probability without normalization. Extended Data Figure 1-2 shows additional parameter results (nontarget and random guess probability).

10.1523/ENEURO.0472-22.2023.f1-2 Figure 1-2 ***A***, Left, Nontarget probability for the Leader (purple) and Follower (turquoise) conditions, with dots denoting individual subjects in experiment 1. Right, Random guess probability in experiment 1. ***B–D***, Same as ***A***, but for experiments 2–4. Correction for multiple comparisons was applied to experiments 3 and 4. Download Figure 1-2, TIF file.

All trials were then categorized based on whether the luminance sequence of the corresponding disk during the delay period (i.e., one with the same color as the probe) was a Leader or Follower sequence, regardless of its color or location. For instance, when recalling the orientation of a red bar held in memory, this trial would be labeled according to whether the luminance sequence of the red disk was a Leader or Follower sequence. Similarly, when retrieving the orientation of the blue bar, the trial condition would be determined by the blue disk, that is, Leader or Follower.

We first estimated memory precision for each item by calculating the reciprocal of the circular SD of response error (the circular difference between the reported orientation and the true orientation across trials, 1∕σ; Bays et al., 2009). As shown in Figure 1*D*, the Leader condition showed better memory performance than the Follower condition (Leader, mean = 1.636, SE = 0.100; Follower, mean = 1.483, SE = 0.111; paired *t* test, *t*_(29)_ = 2.565, *p* = 0.016, Cohen’s *d* = 0.468). We then implemented the Bayesian hypothesis test and confirmed the significant memory modulation effect (BF_10_ = 3.074). To further assess the contribution of the memory for target item to the final report, we used a probabilistic mixture model (Bays et al., 2009) and focused on the calculated target probability, that is, the proportion of responses attributed to the report of the correct target, to quantify memory performance. Moreover, to ensure normal distribution, we performed an empirical logit transformation (de Smith, 2018) on the target response probability. As shown in Fig. 1*E*, the Leader condition also showed better memory performance than the Follower condition (Leader, mean = 3.638, SE = 0.223; Follower, mean = 3.011, SE = 0.220; paired *t* test, *t*_(29)_ = 2.798, *p* = 0.009, Cohen’s *d* = 0.511; Bayes factor, BF_10_ = 4.901; Extended Data Fig. 1-1, target probability without normalization; Extended Data Fig. 1-2, additional parameters, nontarget and random guess probability, results).

Together, consistent with our hypothesis, the Leader–Follower dynamic perturbation during WM retention effectively modulates memory performance when participants held two items in memory, wherein the item experiencing temporal advances during retention shows better memory performance compared with the item with the relative 200 ms temporal delays.

### Memory-irrelevant dynamic perturbation (experiment 2)

In experiment 1, the color feature was memory relevant as participants retained both the orientation and color of the two items. In experiment 2, we examined whether the dynamic perturbation would still be effective when color is memory irrelevant. Thirty new participants participated in experiment 2 (Fig. 2*A*), wherein two bars were simultaneously presented at the top and bottom locations. Instead of memorizing colors as in experiment 1, participants held the locations and orientations of the two bars over a 5 s delay period in memory while performing a central fixation task. During the memory test, participants were first presented with a location cue (top or bottom), and based on this they adjusted a probe bar to match the memorized orientation, regardless of its color. In other words, the color feature was completely memory irrelevant in experiment 2. Similar to experiment 1, the Leader–Follower dynamic perturbation was applied to the two colored disks during retention (Fig. 2*B*,*C*).

Unfortunately, as shown in Figure 2*D*, there is no significant difference between the Leader and Follower conditions on memory precision (Leader, mean = 1.887, SE = 0.628; Follower, mean = 1.920, SE = 0.645; paired *t* test, *t*_(29)_ = −0.460, *p* = 0.649, Cohen’s *d* = −0.084; Bayes factor, BF_10_ = 0.214). Nevertheless, the normalized target probability showed a modulation trend (Leader, mean = 3.454, SE = 0.186; Follower, mean = 3.163, SE = 0.186; paired *t* test, *t*_(29)_ = 1.862, *p* = 0.073, Cohen’s *d* = 0.340; Bayes factor, BF_10_ = 1.012; Fig. 2*E*; Extended Data Fig. 1-1, significant memory modulation effect on target probability). To examine the manipulation consistency between experiments 1 and 2 in terms of the normalized target probability, we conducted a mixed-design ANOVA analysis (Experiment * Perturbation). The results reveal a significant main perturbation effect across experiments, whereas the main effect of the experiments and their interaction effect were nonsignificant (Perturbation effect, *F*_(1,58)_ = 11.288, *p* = 0.001, η_p_^2^ = 0.163; Experiment effect, *F*_(1,58)_ = 0.004, *p* = 0.949, η_p_^2^ < 0.001; Experiment * Perturbation, *F*_(1,58)_ = 1.520, *p* = 0.223, η_p_^2^ = 0.026).This indicates a convergence of evidence from similar experimental designs. The inclusion Bayes factor based on all models further advocates a significant perturbation effect (BF_incl_ = 15.428), and nonsignificant Experiment effect (BF_incl_ = 0.311) and their interaction (BF_incl_ = 0.451).

Overall, the Leader–Follower dynamic perturbation still seems to modulate memory in terms of target probability when the color feature that the dynamic perturbation operates on is memory irrelevant but with a less stronger modulation effect than the memory-relevant perturbation (experiment 1).

### Leader–Follower dynamic perturbation modulates three-item memory performance (experiment 3)

**Figure 3.**
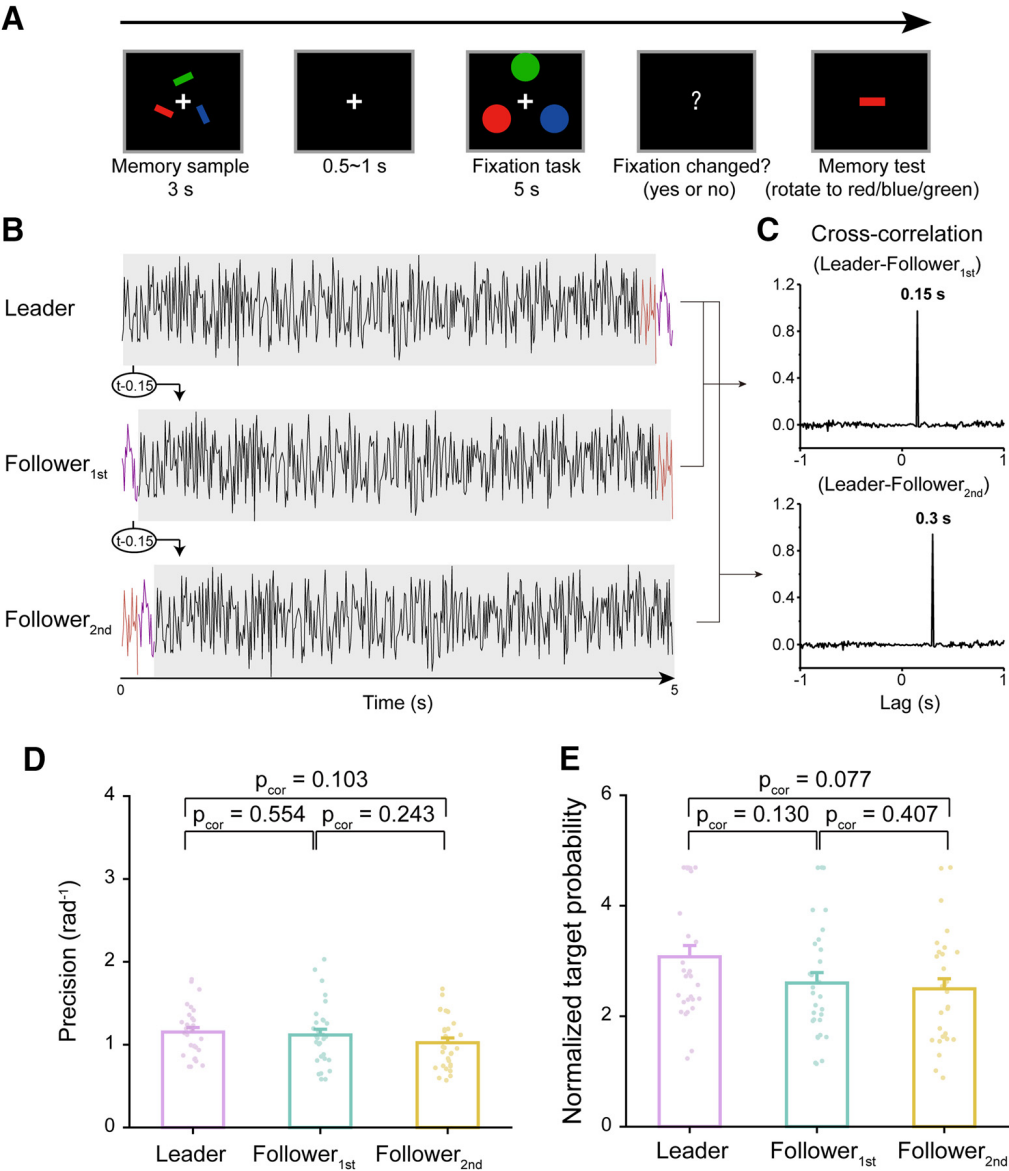
Leader–Follower dynamic perturbation modulates three-item memory performance (experiment 3, *N* = 30). ***A***, Experiment 3 paradigm. In each trial, participants were presented with three bars, and they memorized their orientations and colors. During the memory test, participants adjusted the orientation of a probe bar to match that of the memorized bar that had the same color as the probe. During the 5 s delay period, participants performed a central fixation task while three task-irrelevant flickering disks of the same color as each of the memorized bars (blue, red, green) were presented simultaneously, with their luminance continuously modulated by three 5 s temporal sequences (Leader, Follower_1st_, Follower_2nd_), respectively. The color, spatial location, and Leader–Follower conditions were counterbalanced across trials. ***B***, The Leader temporal sequence was a 5 s white noise randomly generated per trial, and the Follower_1st_ and Follower_2nd_ sequences were created by circular shifting the Leader sequence 150  and 300 ms rightward, respectively. ***C***, The Leader–Follower_1st_ and Leader–Follower_2nd_ cross-correlation over time as a function of temporal lag, peaking at 150  and 300 ms, respectively. ***D***, Memory performance. Grand averaged (mean + SEM) memory precision for Leader (purple), Follower_1st_ (turquoise), and Follower_2nd_ (yellow) conditions. Dots denote individual participants. ***E***, Same as ***D***, but for normalized target probability. Extended Data Figure 1-1 shows target probability without normalization. Extended Data Figure 1-2 shows additional parameter results (nontarget and random guess probability).

After demonstrating the limited effectiveness of the Leader–Follower dynamic perturbation approach in the two-item memory task, we next tested the effectiveness of the approach on a three-item memory display. Thirty new participants participated in experiment 3 (Fig. 3*A*), in which they memorized both the orientations and colors (red, blue, green) of three bars over a 5 s delay period. Similar to experiment 1, during the memory test phase participants adjusted the orientation of a probe bar to match that of the memorized bar with the same color. Critically, the Leader–Follower dynamic perturbation was now applied to three task-irrelevant disks with the same colors as one of the memorized bars (red, blue, green) during the 5 s delay period; their luminance was continuously modulated by three temporally related sequences (Fig. 3*B*). Specifically, one sequence randomly generated in each trial (Leader sequence) was used to generate the other two sequences by introducing a 150 or 300 ms lag, corresponding to the Follower_1st_ and Follower_2nd_ sequences, respectively (Fig. 3*B*,*C*). Using 150  and 300 ms instead of 200 ms derives from previous neural findings revealing that three-item sequence memory entails a more temporally compressed reactivation than the two-item sequence memory (Huang et al., 2018). Finally, the color, spatial location, and Leader–Follower conditions were counterbalanced across trials.

Trials were categorized as Leader, Follower_1st_, or Follower_2nd_ conditions, based on the corresponding luminance sequence (i.e., having the same color as the probe). As shown in Fig. 3*D*, the dynamic perturbation showed weak modulation on memory precision (Leader, mean = 1.153, SE = 0.054; Follower_1st_, mean = 1.117, SE = 0.069; Follower_2nd_, mean = 1.024, SE = 0.058; one-way repeated measures ANOVA; main effect of perturbation, *F*_(2,58)_ = 2.506, *p* = 0.090, η_p_^2^ = 0.080; Bayes factor, BF_incl_ = 0.686; *post hoc* analysis; Leader vs Follower_1st_, *t*_(29)_ = 0.595, *p*_cor_ = 0.554, Cohen’s *d* = 0.107; Bayesian *post hoc* tests, BF_10,U_ = 0.236, posterior odds = 0.138; Leader vs Follower_2nd_, *t*_(29)_ = 2.167, *p*_cor_ = 0.103, Cohen’s *d* = 0.390; Bayesian *post hoc* tests, BF_10,U_ = 1.178, posterior odds = 0.692; Follower_1st_ vs Follower_2nd_, *t*_(29)_ = 1.571, *p*_cor_ = 0.243, Cohen’s *d* = 0.283; Bayesian *post hoc* tests, BF_10,U_ = 0.573, posterior odds = 0.336). Meanwhile, the normalized target probability showed a modulation trend (Leader, mean = 3.077, SE = 0.203; Follower_1st_, mean = 2.599, SE = 0.191; Follower_2nd_, mean = 2.495, SE = 0.184; one-way repeated measures ANOVA; main effect of perturbation, *F*_(2,58)_ = 2.980, *p* = 0.059, η_p_^2^ = 0.093; Bayes factor, BF_incl_ =1.249), revealing a gradual decrease (*post hoc* analysis; Leader vs Follower_1st_, *t*_(29)_ = 1.881, *p*_cor_ = 0.130, Cohen’s *d* = 0.453; Bayesian *post hoc* tests, BF_10,U_ = 0.781, posterior odds = 0.459; Leader vs Follower_2nd_, *t*_(29)_ = 2.288, *p*_cor_ = 0.077, Cohen’s *d* = 0.551; Bayesian *post hoc* tests, BF_10,U_ = 1.424, posterior odds = 0.836; Follower_1st_ vs Follower_2nd_, *t*_(29)_ = 0.407, *p*_cor_ = 0.685, Cohen’s *d* = 0.098; Bayesian *post hoc* tests, BF_10,U_ = 0.216, posterior odds = 0.127).

Together, on a descriptive level, the Leader–Follower dynamic perturbation approach is also effective in a three-item paradigm; that is, the item associated with earlier temporal reactivations shows better memory performance compared with those endowed with relatively delayed reactions during the delay period. However, on a statistical level, the results provide a trend in the suggested direction at best.

### Memory-irrelevant dynamic perturbation in three-item memory task (experiment 4)

**Figure 4.**
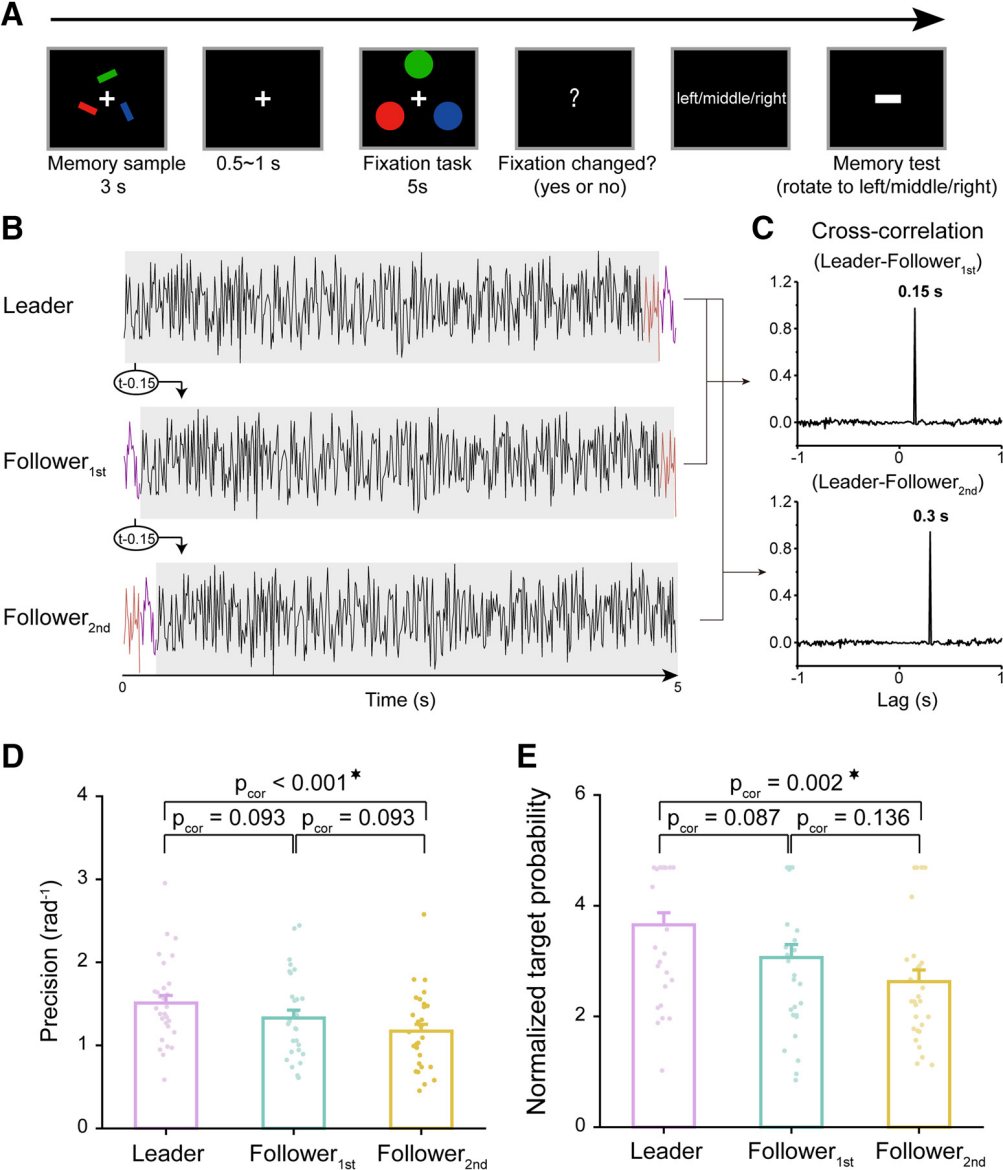
Memory-irrelevant Leader–Follower dynamic perturbation (experiment 4, *N* = 30). ***A***, Task-irrelevant dynamic perturbation paradigm. Experiment 4 was the same as experiment 3, except that participants needed to memorize the orientations and locations (left/middle/right) of the three bar stimuli regardless of their color features. During the memory test period, a location cue was first presented; based on this participants rotated the horizontal white bar to the corresponding memorized orientation. Critically, a Leader–Follower dynamic perturbation as in experiment 3 was applied during the delay period; that is, three disks of the same color as each of the memorized bars (blue, red, green) were presented simultaneously, with their luminance continuously modulated by the Leader, Follower_1st_, or Follower_2nd_ sequence, respectively. ***B***, The Leader temporal sequence was a 5 s white noise randomly generated per trial, and the Follower_1st_ and Follower_2nd_ sequences were created by circular shifting the Leader sequence 150  and 300 ms rightward, respectively. ***C***, The Leader–Follower_1st_ and Leader–Follower_2nd_ cross-correlation over time as a function of temporal lag, peaking at 150  and 300 ms, respectively. ***D***, Memory performance. Grand averaged (mean + SEM) memory precision for Leader (purple), Follower_1st_ (turquoise), and Follower_2nd_ (yellow) conditions. Dots denote individual participants. ***E***, Same as ***D***, but for normalized target probability. Extended Data Figure 1-1 shows target probability without normalization. Extended Data Figure 1-2 shows additional parameter results (nontarget and randomly guess probability).

Finally, we tested the memory-irrelevant dynamic perturbation approach in a three-item memory task (experiment 4). Thirty new participants participated in the experiment (Fig. 4*A*) in which they held the locations and orientations of the three bars over a 5 s delay period in memory. During the memory test phase, participants were first presented with a location cue (left, middle, or right); based on this they adjusted a probe bar to match the memorized orientation, regardless of its color. Thus, similar to experiment 2, the color feature was completely memory irrelevant here. Moreover, the same Leader–Follower dynamic perturbation as used in experiment 3 was applied to the three colored disks during retention (Fig. 4*B*,*C*).

As shown in Figure 4*D*, the memory precision for Leader, Follower_1st_, and Follower_2nd_ conditions exhibited a gradual decrease (Leader, mean = 1.510, SE = 0.091; Follower_1st_, mean = 1.329, SE = 0.094; Follower_2nd_, mean = 1.170, SE = 0.084; one-way repeated measures ANOVA; main effect of perturbation, *F*_(2,58)_ =7.303, *p* = 0.001, η_p_^2^ = 0.201; Bayes factor, BF_incl_ = 22.737; *post hoc* analysis, Leader vs Follower_1st_, *t*_(29)_ = 2.033, *p*_cor_ = 0.093, Cohen’s *d* = 0.368; Bayesian *post hoc* tests, BF_10,U_ = 0.883, posterior odds = 0.519; Leader vs Follower_2nd_, *t*_(29)_ = 3.819, *p*_cor_ < 0.001, Cohen’s *d* = 0.692; Bayesian *post hoc* tests, BF_10,U_ = 40.869, posterior odds = 24.007; Follower_1st_ vs Follower_2nd_, *t*_(29)_ = 1.786, *p*_cor_ = 0.093, Cohen’s *d* = 0.323; Bayesian *post hoc* tests, BF_10,U_ = 1.197, posterior odds = 0.703). The normalized target probability also showed a significant modulation effect (Leader, mean = 3.656, SE = 0.217; Follower_1st_, mean = 3.064, SE = 0.236; Follower_2nd_, mean = 2.630, SE = 0.208; one-way repeated measures ANOVA; main effect of perturbation, *F*_(2,58)_ = 6.435, *p* = 0.003, η_p_^2^ = 0.182; Bayes factor, BF_incl_ = 21.583; *post hoc* analysis; Leader vs Follower_1st_, *t*_(29)_ = 2.062, *p*_cor_ = 0.087, Cohen’s *d* = 0.490; Bayesian *post hoc* tests, BF_10,U_ = 1.101, posterior odds = 0.647; Leader vs Follower_2nd_, *t*_(29)_ = 3.573, *p*_cor_ = 0.002, Cohen’s *d* = 0.849; Bayesian *post hoc* tests, BF_10,U_ = 22.450, posterior odds = 13.187; Follower_1st_ vs Follower_2nd_, paired *t* test, *t*_(29)_ = 1.511, *p*_cor_ = 0.136, Cohen’s *d* = 0.359; Bayes factor, BF_10,U_ = 0.614, posterior odds = 0.360).

To examine the manipulation consistency between experiments 3 and 4, both of which used a three-item WM task, we conducted a mixed-design ANOVA analysis (Experiment * Perturbation) again. The results reveal a significant main perturbation effect across experiments (Perturbation effect, *F*_(2,116)_ = 9.111, *p* < 0.001, η_p_^2^ = 0.136; *post hoc* analysis, Leader vs Follower_1st_, *t*_(58)_ = 2.791, *p*_cor_ = 0.012, Cohen’s *d* = 0.472; Leader vs Follower_2nd_, *t*_(29)_ = 4.193, *p*_cor_ < 0.001, Cohen’s *d* = 0.708; Follower_1st_ vs Follower_2nd_, *t*_(29)_ = 1.401, *p*_cor_ = 0.164, Cohen’s *d* = 0.237; Experiment effect, *F*_(1,58)_ = 4.202, *p* = 0.045, η_p_^2^ = 0.068; Experiment * Perturbation, *F*_(2,116)_ = 0.726, *p* = 0.486, η_p_^2^ = 0.006), supporting the modulation effect across experiments. The inclusion Bayes factor based on all models further advocates a significant perturbation effect (BF_incl_ = 134.346; *post hoc* tests, Leader vs Follower_1st_, BF_10,U_ = 3.748, posterior odds = 2.202; Leader vs Follower_2nd_, BF_10,U_ = 133.865, posterior odds = 78.633; Follower_1st_ vs Follower_2nd_, BF_10,U_ = 0.435, posterior odds = 0.256), whereas the main effect of Experiment (BF_incl_ = 0.823) and the interaction effect (BF_incl_ = 0.376) were nonsignificant. Overall, the Leader–Follower dynamic perturbation efficiently modulates three-item memory when the color feature that the dynamic perturbation operates on is memory irrelevant.

To provide a possible explanation for the nonrobust memory modulation effect in experiments 2 and 3, we compared the memory precision between experiments, which we thought should largely reflect the task difficulty. Experiment 2 (two-item location memory) showed significant higher memory precision compared with experiment 1(two-item color memory; Experiment effect, *F*_(1,58)_ = 5.264, *p* = 0.025, η_p_^2^ = 0.083; BF_incl_ = 2.191), whereas experiment 4 (three-item location memory) also showed significant higher memory precision than experiment 3 (three-item color memory; Experiment effect, *F*_(1,58)_ = 7.242, *p* = 0.009, η_p_^2^ = 0.111; BF_incl_ = 5.030). These results indicated that this purely bottom-up perturbation may only have significant effectiveness when the task is of moderate difficulty instead of being too easy (two-item location memory) or too difficult (three-item color memory) to accomplish.

## Discussion

In the present study, we sought to capitalize on the Leader–Follower dynamic perturbation as a new behavioral manipulation mechanism to interfere with the multi-item neural dynamics and alter WM performance in humans. Four experiments with 120 participants demonstrate the effectiveness of the approach. Specifically, temporally advanced manipulation (Leader) during retention leads to better recalling performance than temporally delayed perturbation (Follower), regardless of its relevance to the memory task. These findings, together with previous works (Miller et al., 2018; Barbosa et al., 2020; Li et al., 2021), support the substantial role of STP-based neural dynamics in mediating WM operation. Our work also offers a new bottom-up behavioral approach to manipulating human WM. However, it is notable that the memory modulation effect is not very robust across experiments and measures, which indicates that this purely bottom-up perturbation approach has limited effectiveness and needs further exploration.

There are many noninvasive approaches to altering WM performance in humans. For instance, applying TMS to relevant brain regions could modulate memory behavior (Lee and D’Esposito, 2012) and even reactivate information retained in WM (Rose et al., 2016). Oscillatory interference methods, such as rhythmic physical stimulus (Clouter et al., 2017), repetitive TMS (Sauseng et al., 2009; Beynel et al., 2019), or tACS with rhythmic (Hoy et al., 2015) or theta–gamma coupling (Alekseichuk et al., 2016), have also been found to efficiently affect memory performance. Here, we developed a purely bottom-up, behavioral approach by presenting task-irrelevant flickering color probes during WM retention. Notably, because participants could not discriminate the temporal relationship of the luminance sequences at the perceptual level, that is, which sequence leads and which sequence lags, the manipulation is indeed operated in an unconscious way. Moreover, the luminance sequences are randomly generated per trial, and therefore it is only their temporal relationship instead of a specific sequence that influences WM performance. Furthermore, the Leader–Follower dynamic perturbation aims to alter multi-item WM performance, which is different from our previous work focusing on sequence working memory (Li et al., 2021), thus offering a memory manipulation approach at a general level. Finally, distinct from the retrocue behavioral paradigm, whereby the cued item would enter the focus of attention and get prioritized in WM (Oberauer and Hein, 2012; Oztekin et al., 2010), our method is a purely bottom-up manipulation and does not rely on top-down attentional modulations.

Crucially, our Leader–Follower dynamic perturbation approach draws on accumulating findings and models advocating the central function of temporal dynamics in WM. First, multiple WM items are postulated to undergo item-by-item sequential reactivations with items of greater strength firing earlier (Lisman and Idiart, 1995; Oberauer and Lewandowsky, 2008, 2011; Lisman and Jensen, 2013), a framework that has received empirical evidence support (Axmacher et al., 2010; Friese et al., 2013; Burke et al., 2016; Heusser et al., 2016). Recently, we also demonstrate that a sequence of items is serially reactivated during the delay period, and the late item in the sequence is accompanied by better memory performance (i.e., recency effect) and earlier reactivation (Huang et al., 2018, 2021), also in line with the latency-based view. Interestingly, this latency- or time-based coding of input strength extends beyond memory findings and also occurs in perception and attention (Landau and Fries, 2012; Fiebelkorn et al., 2013, 2018; Jensen et al., 2014; Song et al., 2014; Jia et al., 2017; Mo et al., 2019; Huang and Luo, 2020). Here, we speculate that altering the early–late time relationship of neural responses indeed modifies the subsequent WM performance. Second, the time lag between luminance sequences is set also according to previous experimental findings and STP neural model, that is, temporally compressed reactivation within 200  and 150 ms for two- and three-item sequences, respectively (Herweg et al., 2020; Huang et al., 2018; Li et al., 2021; Mi et al., 2017; Mongillo et al., 2008). Overall, the dynamic perturbation approach is motivated by previous findings, allowing us to exploit the time perspective of the brain to manipulate multi-item neural dynamics and in turn alter WM performance.

We developed a Leader–Follower dynamic perturbation aiming to introduce a specific temporal lag in the reactivation profiles of memorized items to manipulate their memory strengths. We hypothesize that items with relatively earlier reactivation during retention would have better memory performance than items with relatively later reactivation. The manipulation is implemented by generating temporally shifted luminance sequences (i.e., Leader sequence, Follower sequence) for color disks that are bound to each memorized item during retention. Although the temporal manipulation is possibly at an unconscious level, that is, participants could not tell which sequence advances over time, our brain is known to be indeed endowed with tremendous capabilities to calculate the temporal lag between events, from tens of milliseconds to hundreds of milliseconds. Moreover, the continuous attractor neural network model established in our previous work, by incorporating plausible biological principles, also supports that temporal lag is encoded in the system and influences memory representations (Li et al., 2021).

Retaining information in WM has traditionally been hypothesized to rely on persistent firing, but computational models and recent findings propose a hidden-state WM view; that is, items could be silently retained in STP-based synaptic weights (Huang et al., 2021; Miller et al., 2018; Mongillo et al., 2008; Rose et al., 2016; Trübutschek et al., 2019; Wolff et al., 2017), even lasting for tens of seconds long with a periodical refresh (Fiebig and Lansner, 2017). Then how could we access information in this activity-silent network? Previous studies demonstrate that presenting a nonspecific impulse during retention could transiently perturb the WM network and reactivate memories (Fan et al., 2021; Huang et al., 2021; Wolff et al., 2017). This methodological advance has allowed researchers to directly access WM information and predict subsequent behavior. Here, we use task-irrelevant luminance sequences to first reactivate memory information and then apply continuous perturbation to impose temporal relationships between items to interfere with their neural dynamics and manipulate WM. This approach resembles the reconsolidation process in long-term memory such that the stored fear memory would be rendered labile when retrieved, and new information could be inserted and modify old memories within this period (Agren et al., 2012; Lane et al., 2015; Schiller et al., 2010). Meanwhile, different from long-term memory relying on long timescales, our approach is operated at a shorter temporal scale, that is, 100–200 ms, a critical time scale in STP-based WM operation.

Together, based on accumulating neural findings and theoretical models, we develop a new Leader–Follower dynamic perturbation behavioral approach to alter multi-item WM in humans by presenting temporally related luminance sequences during the delay period. We demonstrate that the item associated with the Leader luminance sequence shows better memory performance than the item bound to the Follower luminance sequence. Our results suggest the essential role of neural temporal dynamics in WM operation and offer a promising, noninvasive WM manipulation approach.

## Synthesis

Reviewing Editor: Anne Keitel, University of Dundee Decisions are customarily a result of the Reviewing Editor and the peer reviewers coming together and discussing their recommendations until a consensus is reached. When revisions are invited, a fact-based synthesis statement explaining their decision and outlining what is needed to prepare a revision will be listed below. The following reviewer(s) agreed to reveal their identity: NONE. Note: If this manuscript was transferred from JNeurosci and a decision was made to accept the manuscript without peer review, a brief statement to this effect will instead be what is listed below.

The reviewers and reviewing editor agreed that the study is novel and potentially interesting. However, based on the reviewer comments, these are the main points that need to be addressed in a revised version of the manuscript (unabridged reviewer comments below):

1) Methodology: There are some issues with the statistics, for example no correction for multiple comparisons, and interpreting “marginally significance” as significant.

2) Clarifications/explanations: The rationale and hypotheses, as well as methods, should be described clearer.

You will find these main issues, as well as other comments below. I’m attaching the detailed reviewer comments so that they can be addressed in a point-by-point manner:

Reviewer #1 - Advances the Field (Required)

The approach described in the manuscript has the potential to advance the field, by providing a new methodologial approach to manipulating the strength of working memory representations. However, as outlined in my comments, despite the series of experiments that were conducted, I am not entirely convinced that the data support the authors conclusions.

Reviewer # 1 - Statistics

The authors frequently interpret the p-value as a continuous measure of evidence, which is not valid. Further, non-significant p-values in-between .05 and .10 (referred to as marginally significant by the authors) are simply interpreted as if significance, in line with the authors hypotheses.

Reviewer #1 - Comments to the Authors

Summary: The present manuscript is based on a series of five experiments with different variants of a multi-item working memory paradigm. Participants were asked to maintain the orientation and one additional feature (e.g., color or spatial location) of either two or three colored bars. At the end of a trial, the orientation of a probe stimulus had to be adjusted to match the orientation of the cued memory item. Critically, during the maintenance period, two colored disks were presented with continuously varying luminance (i.e., luminance was continuously modulation to create the impression of two flickering disks). The temporal relationship was of the luminance sequences was modulated in a way that one sequence lagged behind. The authors refer to this as the “Leader-Follower” approach. Across experiments, memory performance for the item associated with the leading luminance sequence was better than that of the follower-sequence. This was the case irrespective of whether color (i.e., the feature that links the perturbation to the memory items) was task-relevant or not and irrespective of memory load (two versus three items).

Overall assessment: The present approach to modulate the representational strength of working memory items is indeed interesting and innovative. Yet, currently I think the manuscript falls short of its potential. One major reason why it doesn’t convince me is that currently the underlying rational of the perturbation approach, the assumed mechanisms and the hypotheses poorly described. More critically, across all experiments, some of the critical effects fall short of the criterion of significance (p = .05) but are nonetheless interpreted as significant. Overall, this raises some doubts about how well the data really support the conclusions and requires a critical re-assessment of the results by the authors. I provide more detailed comments and questions below.

Major comments:

- Just based on the introduction, the underlying rational of the “Leader-Follower” approach is hard to understand. I know that the journal limits the introduction to 750 words, but I really think the rational needs to be explained more thoroughly. For instance, it might be beneficial to introduce the previous work by Li et al (2021) in more detail to illustrate the principle of how the flickering disks are assumed to influence working memory. From what I understood, the general idea is that the flickering disks presented in the memory interval have a similar function as the “ping” in the study by Wolff et al 2017, reactivating memories from an activity-silent state. But, in contrast to the Wolff approach, where the ping is a relatively unspecific stimulus, here, the idea is that because the colors of the flickering disks match one of the memory items, they specifically modulate the neural activity associated with the maintenance of that particular item. Is that correct? What is still not clear to me though is: Why are the items assumed to be an activity-silent state rather than actively maintained state in the first place? Is it because participants are engaged in the central fixation task, rendering the memory items temporarily task-irrelevant? Because the rhythms of the two flickering disks differ, you assume that the memory items are going to be reactivated in a different temporal order, right? Can you elaborate more specifically on what mechanism you assume is underlying this modulation of temporal relationships? I feel like simply mentioning the “serial reactivation” idea (p.4, lines 62-65), not sufficient.

- Specifically, what should be elaborated in more detail are the hypotheses underlying the different manipulations used across experiments. Moreover, based on what rational would you expect that the “leading sequence” results in better memory performance? This should be outlined more explicitly.

- I am not sure I understand the concept of the “temporal advances”. I assume, the rational is that the item associated with the leading sequence is reactivated earlier. However, I don’t quite understand how the brain is supposed to know which one of the luminance sequences is “leading” as opposed to “lagging”. The sequences are generated randomly and have no particular meaning. You could have also taken the first 200 ms of one sequence and copied it to the end of the sequence (instead of taking the last 200 ms of one sequence and moving it to the beginning of the sequence) - wouldn’t that reverse the whole thing while having the same degree of temporal correlation? In other words, why does taking something off the end of a sequence and attaching it to the beginning make it the follower sequence?

- Only after reading the methods and results it became clear to my how your experiments are also linked to the theory of “object-based WM” (p. 5, lines 93-94) - but just based on the introduction it wasn’t really clear how that theory motivated your approach. If the authors outline their hypotheses and the related experimental manipulations explicitly, this will be easier to understand.

- P.6, lines 115-117: The authors indicate that sample size was determined based on a pretest on 25 subjects, using a similar paradigm as in Experiment 2 and cite the software g-power along with this statement. If a formal a-priori power analysis was performed, this should be described in more detail. What was the expected effect size used for the power analysis? What power did you aim for? Also, at least until a couple of years ago, g-power did not support power analysis for repeated-measures ANOVA with more than one factor. As some of the analyses in the present manuscript use a two-way repeated measures ANOVA, this should be considered. I recommend using either MorePower6.0 (Campbell & Thompson, 2012: https://link.springer.com/article/10.3758/s13428-012-0186-0) to verify the calculations performed in g-power.

- Given that the underlying mechanisms of the present approach remain speculative (i.e., are not measured on a neural basis), can you really argue for causal evidence here ? (e.g., p. 18, lines 447-448)

- P. 12, lines 286-287: do not use the term “marginally significant” for p-values >.05 and < .10. Under the null hypothesis, p-values are uniformly distributed. Hence, a p-value of .07 is just as likely as a p-value of .80. In contrast, if the alternative hypothesis is true, the strength of evidence that corresponds to the p-value depends on the statistical power of the test. By using the term “marginally significant”, you imply that the p-value can be interpreted as the strength of evidence. That this is not valid is illustrated by the Jeffrey-Lindley paradox, which basically illustrates that the same p-value can correspond to different levels of relative evidence (for a nice illustration of this phenomenon, see this blog post: https://daniellakens.blogspot.com/2021/11/why-p-values-should-be-interpreted-as-p.html).

- Adding to the above comment, I have to say that I have the impression, that the authors apply that logic of using the p-value as a continuous measure of evidence quite a lot and interpret their findings in favor of their hypotheses without critically evaluating the statistical results. P-values that barely fall short of the .05-cutoff (i.e., >.05 but >.10) are simply considered significant if that suits the expected pattern: For instance, in experiment 1, the leader-follower main effect (in the rm-anova including location as a factor) is not significant (p = .07) - yet, the effect is described as being “effective for both upper and lower locations of the memorized item”. In Experiment 2, a p-value of .07 is considered as “marginally significant”. In Experiment 3, the main effect of perturbation is also not significant (p = .07 if correctly rounded - according to statcheck.io, see comment below), yet, follow-up paired sample t-tests are conducted. The latter also result in mixed results, with the leader vs. follower-1 comparison yielding a p-value of .09. Again, despite above-threshold p-values, the authors generalize the results as reflecting the successful modulation of three-item memory performance. Similar generalized conclusions are drawn in experiment 4, where leader vs. follower-1 is not significant (p = .06) while leader vs. follower-2 is significant (p = .002). Finally, none of the follow-up paired-sample t-tests are corrected for multiple comparisons. Overall, this raises some doubts about the correctness of the statistical analyses, the statistical power of the experiments and whether the conclusions are really well-supported by the data. I recommend that the authors critically re-assess their interpretations and conclusions obtained from the data.

Minor comments:

- I find it confusing that the colored disks presented in the maintenance interval are referred to as “probes” throughout the manuscript. In the working memory literature, the term “probe” is typically linked with the recall phase of a task and denotes that stimulus that cues a particular item for recall. To avoid confusion, I recommend referring to the stimuli as colored disks.

- Based on the description of the paradigm, it is not clear to me what the fixation task entails. On p. 7 you indicate that subjects were asked to “monitor an abrupt luminance change”. However, as far as I understood, the luminance of the colored disks presented during the maintenance period ALWAYS changed. If that was the case, the task is redundant, isn’t it? According to the figures, though, subjects were asked to indicate whether “fixation changed”? This sounds like something different. Could you clarify this?

- Please report the RGB values associated with the red and blue color used in the experiments.

- Figure 1D: leader and follower should be labeled as such (instead of just using “B” and “C” as labels on the x-axis).

- Statcheck.io (https://michelenuijten.shinyapps.io/statcheck-web/) identified two inconsistencies in your reported statistical results. Please check the p-values for the main effects of perturbation condition on p.13 (line 337) and p.15 (line 378).

- In experiment 3 and 4, the comparison between follower 1 and follower 2 are not significant. Was this to be expected? Why? Both follower 1 and follower are derived from the same sequence, so shouldn’t they also be temporally correlated?

- For the sake of completeness, I think the additional parameters estimated by the mixture model (probability of reporting a non-target and probability of responding randomly) should also be provided as supplementary material.

Reviewer #2 - Advances the Field

If actually proven effective, the authors demonstrate an interesting and novel approach to subtly manipulate the performance of individual items in multi-item WM tasks

Reviewer #2 - Statistics

The authors do not correct for multiple comparisons: For example, the rANOVA main effect in question (containing three levels) is not significant for Experiment 3 and the follow-up pairwise comparisons are not corrected, yet the authors conclude that there is in an effect.

Reviewer #2 - Comments to the Authors

The authors demonstrate a novel and interesting approach to manipulate the performance in a multi-item WM task. I have a few concerns about the methodology, statistical analyses, and the conclusions drawn, however.

Major:

1. The authors use target probability from the mixture model as the dependent measure for all main analyses, instead of absolute error or another parameter from the model (i.e. SD). They justify doing so by giving references of other works (line 205). However, apart from their own previous work, the given references actually reported all parameters, as well as raw performance. Indeed, this is also what the authors should do here. In fact, it seems that the number of trials is insufficient to properly model the response distributions, as can be seen in the normalized target probability plots where the performance of many subjects seems to be at the peak of the distribution (at around 5), suggesting that the mixture model estimated a guess-rate of exactly 0 for those subjects. I thus strongly suggest not to use the model on this data, or, at the very least acknowledge the shortcomings and explicitly report the same analyses on the other parameters (most notably SD) and raw performance (this should be moved from the supplemental to the main text and figures).

2. The authors should acknowledge that many of the results are weak/absent and not statistically significant. Experiment 2 shows a non-significant trend of an effect on the normalized target probability. Figure S2B, suggests that when actually looking at the raw performance, the effect, if anything, might actually be the other way round. I wonder what the SD parameter looks like here. Furthermore, the paired t-tests in Experiments 3&4 should be corrected for multiple comparisons. When done, then only two out of the four experiments have statistically significant effects. I understand that the significance threshold is arbitrary. I suggest complementing all frequentist statistics with Bayesian statistics.

Minor:

1. What does B and C stand for in the bar plots?

2. Was the mixture modelling done separately within each sub-condition (i.e. upper and leader, lower and leader, and so on?) How many trials were used for each model?

3. Line 207: The authors state that “p” is target probability in the formula. Is that really so? Isn’t it the inverse, that is, guess rate?

4. In Figure S1B a control Experiment is reported, which is not further acknowledged in the main text. Also, what was the a priori reason to suspect that 500 ms would be too long of a time-lag to be effective?

5. In lines 423 to 425 the authors state that the manipulation was unconscious and that subjects could not discriminate the sequences. Was this actually tested? -

## Author Response

Synthesis Statement for Author (Required):

The reviewers and reviewing editor agreed that the study is novel and potentially interesting. However, based on the reviewer comments, these are the main points that need to be addressed in a revised version of the manuscript (unabridged reviewer comments below):

1) Methodology: There are some issues with the statistics, for example no correction for multiple comparisons, and interpreting “marginally significance” as significant.

2) Clarifications/explanations: The rationale and hypotheses, as well as methods, should be described clearer.

You will find these main issues, as well as other comments below. Please use the detailed reviewer comments to address each comment in a point-by-point manner:

Reviewer #1 - Advances the Field (Required)

The approach described in the manuscript has the potential to advance the field, by providing a new methodological approach to manipulating the strength of working memory representations. However, as outlined in my comments, despite the series of experiments that were conducted, I am not entirely convinced that the data support the authors conclusions.

Reviewer # 1 - Statistics

The authors frequently interpret the p-value as a continuous measure of evidence, which is not valid. Further, non-significant p-values in-between .05 and .10 (referred to as marginally significant by the authors) are simply interpreted as if significance, in line with the authors hypotheses.

Reviewer #1 - Comments to the Authors

Summary: The present manuscript is based on a series of five experiments with different variants of a multi-item working memory paradigm. Participants were asked to maintain the orientation and one additional feature (e.g., color or spatial location) of either two or three colored bars. At the end of a trial, the orientation of a probe stimulus had to be adjusted to match the orientation of the cued memory item. Critically, during the maintenance period, two colored disks were presented with continuously varying luminance (i.e., luminance was continuously modulation to create the impression of two flickering disks). The temporal relationship was of the luminance sequences was modulated in a way that one sequence lagged behind. The authors refer to this as the “Leader-Follower” approach. Across experiments, memory performance for the item associated with the leading luminance sequence was better than that of the follower-sequence. This was the case irrespective of whether color (i.e., the feature that links the perturbation to the memory items) was task-relevant or not and irrespective of memory load (two versus three items).

Overall assessment: The present approach to modulate the representational strength of working memory items is indeed interesting and innovative. Yet, currently I think the manuscript falls short of its potential. One major reason why it doesn’t convince me is that currently the underlying rational of the perturbation approach, the assumed mechanisms and the hypotheses poorly described. More critically, across all experiments, some of the critical effects fall short of the criterion of significance (p = .05) but are nonetheless interpreted as significant. Overall, this raises some doubts about how well the data really support the conclusions and requires a critical re-assessment of the results by the authors. I provide more detailed comments and questions below.

We thank the reviewer for the important concerns. Please see our point-to-point response as below.

Major comments:

- Just based on the introduction, the underlying rational of the “Leader-Follower” approach is hard to understand. I know that the journal limits the introduction to 750 words, but I really think the rational needs to be explained more thoroughly. For instance, it might be beneficial to introduce the previous work by Li et al (2021) in more detail to illustrate the principle of how the flickering disks are assumed to influence working memory. From what I understood, the general idea is that the flickering disks presented in the memory interval have a similar function as the “ping” in the study by Wolff et al 2017, reactivating memories from an activity-silent state. But, in contrast to the Wolff approach, where the ping is a relatively unspecific stimulus, here, the idea is that because the colors of the flickering disks match one of the memory items, they specifically modulate the neural activity associated with the maintenance of that particular item. Is that correct? What is still not clear to me though is: Why are the items assumed to be an activity-silent state rather than actively maintained state in the first place? Is it because participants are engaged in the central fixation task, rendering the memory items temporarily task-irrelevant? Because the rhythms of the two flickering disks differ, you assume that the memory items are going to be reactivated in a different temporal order, right? Can you elaborate more specifically on what mechanism you assume is underlying this modulation of temporal relationships? I feel like simply mentioning the “serial reactivation” idea (p.4, lines 62-65), not sufficient.

We thank the reviewer for pointing out the unclear rationale and motivation for the dynamic perturbation approach and we apologize for the lack of clarity. The reviewer is correct that this approach is motivated by the “PING” approach but still differs from each other, i.e., item-specific vs. nonspecific. Meanwhile, we would like to emphasize that the dynamic perturbation approach does not rely on the activity-silent assumption. Instead, our major motivation is that no matter whether memory information is stored in an active or activity-silent way, information needs to be in an active state to be manipulated. Crucially, our previous study revealed that memory-related flickering probes could tag item-specific neural reactivations during memory retention (Huang et al., eLife, 2018), which motivates our idea of using flickering probes to interfere with item-specific reactivations to manipulate WM performance. This is exactly the rationale behind the dynamic perturbation approach (Li et al., Progress in Neurobiology, 2021).

With regards to the temporal relationship imposed on memorized items, first, note that all the luminance sequences are just white noise instead of a specific rhythm. Crucially, we introduced a systematic temporal lag between the luminance sequences of the two color probes so that their cross-time correlation is fixed at a certain time, e.g., 200 ms (Fig. 1C and 2C). This could be regarded as that one sequence, although as a random white noise that does not contain any regularities, always precedes another random sequence by 200 ms. Correspondingly, the reactivations for the two memorized items that are presumably bound to the flickering color probes would also have 200 ms of temporal lag. In other words, the item bound to the leader luminance sequence would have temporally advanced reactivations compared to the item associated with the follower luminance sequence.

Clarifications have been added now in Introduction, Methods, and Discussion.

- Specifically, what should be elaborated in more detail are the hypotheses underlying the different manipulations used across experiments. Moreover, based on what rational would you expect that the “leading sequence” results in better memory performance? This should be outlined more explicitly.

Hypotheses have been added now.

The rationale behind the hypothesis that leading sequences will be associated with better memory performance is based on previous neural evidence and theoretical frameworks. Specifically, the item with stronger memory strength, given its higher neural excitability, fires at an earlier latency, while the less excitable item reactivates relatively late (Bahramisharif et al., 2018; Huang et al., 2018, 2021; Siegel et al., 2009). As a consequence, we hypothesize that temporally advanced sequence during retention would facilitate subsequent memory performance compared to the temporally delayed sequence.

More elaborations have been added in Introduction.

- I am not sure I understand the concept of the “temporal advances”. I assume, the rational is that the item associated with the leading sequence is reactivated earlier. However, I don’t quite understand how the brain is supposed to know which one of the luminance sequences is “leading” as opposed to “lagging”. The sequences are generated randomly and have no particular meaning. You could have also taken the first 200 ms of one sequence and copied it to the end of the sequence (instead of taking the last 200 ms of one sequence and moving it to the beginning of the sequence) - wouldn’t that reverse the whole thing while having the same degree of temporal correlation? In other words, why does taking something off the end of a sequence and attaching it to the beginning make it the follower sequence?

Sorry for the unclear description. As explained previously, the temporal advance refers to the systematic time lag between two temporal sequences so that their cross-correlation is fixed at a certain value, e.g., 200 ms (Fig. 1C and 2C). In other words, the leader sequence, although being a white noise that does not contain any regularities, always precedes another random sequence by 200 ms. As the reviewer states, we hypothesize that this temporal manipulation will result in the item with leader sequence reactivating earlier than the item with follower sequence. Meanwhile, the early reactivation is not just due to the copy of the last 200 ms to the beginning of another sequence or the other way around. In fact, the temporal relationship between the two luminance sequences is ongoing and continuous throughout the whole delay period, since the leader sequence always precedes the follower sequence by 200 ms. The reason we copied the last 200 ms segment of one sequence to the beginning of another sequence is to make sure that the two temporal sequences occur simultaneously while at the same time having a 200 ms fixed time lag. In other words, to make two sequences with fixed time lag, we temporally shifted one sequence (leader sequence) rightward by 200 ms to generate the follower sequence; to make the two temporal sequences with the same onset, we then cut the last 200 ms segment of the follower sequence and shift it to its beginning so that the two sequences occur simultaneously.

The reviewer is correct that we could also take the first 200 ms of the original sequence and copy it to the end of the sequence to generated a new sequence. In this case, the original sequence would lag the new sequence by 200 ms, and as a result, the original sequence would be the “Follower”, while the new sequence would be the “Leader”.

Regarding how our brains know which one of the luminance sequences is “Leader” or “Follower”, we posit that the brain is endowed with capabilities to calculate the temporal lag between events, from tens of milliseconds to hundreds of milliseconds. In fact, our hearing system relies on temporal lag to calculate the auditory source. Moreover, the neural network model in our previous work also supports that temporal lag information between items could be represented in the system and contribute to memory representation (Li et al., 2021).

We thank the reviewer for the important question and have added more clarification in Introduction, Methods and Discussion.

- Only after reading the methods and results it became clear to my how your experiments are also linked to the theory of “object-based WM” (p. 5, lines 93-94) - but just based on the introduction it wasn’t really clear how that theory motivated your approach. If the authors outline their hypotheses and the related experimental manipulations explicitly, this will be easier to understand.

Thanks for the suggestion. Added now.

- P.6, lines 115-117: The authors indicate that sample size was determined based on a pretest on 25 subjects, using a similar paradigm as in Experiment 2 and cite the software g-power along with this statement. If a formal a-priori power analysis was performed, this should be described in more detail. What was the expected effect size used for the power analysis? What power did you aim for? Also, at least until a couple of years ago, g-power did not support power analysis for repeated-measures ANOVA with more than one factor. As some of the analyses in the present manuscript use a two-way repeated measures ANOVA, this should be considered. I recommend using either MorePower6.0 (Campbell & Thompson, 2012: https://link.springer.com/article/10.3758/s13428-012-0186-0) to verify the calculations performed in g-power.

Thanks for this suggestion. The expected effect size is around 0.55 and the aimed power is 0.8, which result in a sample size of 28. We thank the reviewer for the recommendation of MorePower6.0, and we indeed obtained similar sample size using MorePower6.0 and Gpower.

We have added related clarification in Methods.

- Given that the underlying mechanisms of the present approach remain speculative (i.e., are not measured on a neural basis), can you really argue for causal evidence here? (e.g., p. 18, lines 447-448)

Thanks for raising this concern. Modified now.

- P. 12, lines 286-287: do not use the term “marginally significant” for p-values >.05 and < .10. Under the null hypothesis, p-values are uniformly distributed. Hence, a p-value of .07 is just as likely as a p-value of .80. In contrast, if the alternative hypothesis is true, the strength of evidence that corresponds to the p-value depends on the statistical power of the test. By using the term “marginally significant”, you imply that the p-value can be interpreted as the strength of evidence. That this is not valid is illustrated by the Jeffrey-Lindley paradox, which basically illustrates that the same p-value can correspond to different levels of relative evidence (for a nice illustration of this phenomenon, see this blog post: https://daniellakens.blogspot.com/2021/11/why-p-values-should-be-interpreted-as-p.html).

Thanks for your comments. According to the reviewer’s suggestion, we have weakened our interpretation on the p-values in Revision. Moreover, we would like to emphasize the consistent findings across experiments in our study. Moreover, to resolve the Jeffrey-Lindley paradox, we have implemented the Bayesian statistics, and observed consistent results of frequentist statistic test (see Results).

- Adding to the above comment, I have to say that I have the impression, that the authors apply that logic of using the p-value as a continuous measure of evidence quite a lot and interpret their findings in favor of their hypotheses without critically evaluating the statistical results. P-values that barely fall short of the .05-cutoff (i.e., >.05 but >.10) are simply considered significant if that suits the expected pattern: For instance, in experiment 1, the leader-follower main effect (in the rm-anova including location as a factor) is not significant (p = .07) - yet, the effect is described as being “effective for both upper and lower locations of the memorized item”. In Experiment 2, a p-value of .07 is considered as “marginally significant”. In Experiment 3, the main effect of perturbation is also not significant (p = .07 if correctly rounded - according to statcheck.io, see comment below), yet, follow-up paired sample t-tests are conducted. The latter also result in mixed results, with the leader vs. follower-1 comparison yielding a p-value of .09. Again, despite above-threshold p-values, the authors generalize the results as reflecting the successful modulation of three-item memory performance. Similar generalized conclusions are drawn in experiment 4, where leader vs. follower-1 is not significant (p = .06) while leader vs. follower-2 is significant (p = .002). Finally, none of the follow-up paired-sample t-tests are corrected for multiple comparisons. Overall, this raises some doubts about the correctness of the statistical analyses, the statistical power of the experiments and whether the conclusions are really well-supported by the data. I recommend that the authors critically re-assess their interpretations and conclusions obtained from the data.

Sorry for the inconsistency. Following the reviewer’s suggestions, we have largely revised our interpretations. Moreover, to confirm the reliability of our results, we did the following new analysis. Firstly, as suggested by reviewer 2, we reported the raw performance of target probability (see Fig.1-4 C). Secondly, we performed a two-sample repeated ANOVA across experiments (Experiment (Experiment 1 vs. Experiment 2) * perturbation modulation (Leader vs. Follower) : F(1,58) = 1.52, p = 0.22, ηp2 = 0.03; perturbation modulation: F(1,58) = 11.29, p = 0.001, ηp2 = 0.16; Experiment (Experiment 3 vs. Experiment 4) * perturbation modulation: F(2,58) = 0.73, p = 0.49, ηp2 = 0.01; perturbation modulation: F(2,58) = 9.11, p < 0.001, ηp2 = 0.14). Finally, we have also implemented the Bayesian statistic (BF10 = 20.88 for Experiment 1&2, and BF10 = 180.6 for Experiment 3&4). Together, we hope the new analyses could strengthen the conclusions.

Minor comments:

- I find it confusing that the colored disks presented in the maintenance interval are referred to as “probes” throughout the manuscript. In the working memory literature, the term “probe” is typically linked with the recall phase of a task and denotes that stimulus that cues a particular item for recall. To avoid confusion, I recommend referring to the stimuli as colored disks.

Thanks for this suggestion. Modified.

- Based on the description of the paradigm, it is not clear to me what the fixation task entails. On p. 7 you indicate that subjects were asked to “monitor an abrupt luminance change”. However, as far as I understood, the luminance of the colored disks presented during the maintenance period ALWAYS changed. If that was the case, the task is redundant, isn’t it? According to the figures, though, subjects were asked to indicate whether “fixation changed”? This sounds like something different. Could you clarify this?

Sorry for the confusion. What participants should do is monitoring an abrupt luminance change of the central fixation, not the color disks. Clarified now.

- Please report the RGB values associated with the red and blue color used in the experiments.

The luminance of the flickering discs was first randomly selected (ranging from 0 cd/m2 to 15 cd/m2) and then tailored to have equal power at all frequencies. The luminance was next transferred to RGB values separately for red and blue colors, based on gamma corrections.

Added in Methods.

- Figure 1D: leader and follower should be labeled as such (instead of just using “B” and “C” as labels on the x-axis).

Corrected.

- Statcheck.io (https://michelenuijten.shinyapps.io/statcheck-web/) identified two inconsistencies in your reported statistical results. Please check the p-values for the main effects of perturbation condition on p.13 (line 337) and p.15 (line 378).

Sorry for the mistakes. Corrected now.

- In experiment 3 and 4, the comparison between follower 1 and follower 2 are not significant. Was this to be expected? Why? Both follower 1 and follower are derived from the same sequence, so shouldn’t they also be temporally correlated?

The reviewer is correct that the Follower1st and Follower2nd are temporally correlated and are therefore expected to show a difference. Unfortunately, we only observed a nonsignificant trend. This might be due to the low signal-to-noise ratio for the 3-item sequence. Added in Results.

- For the sake of completeness, I think the additional parameters estimated by the mixture model (probability of reporting a non-target and probability of responding randomly) should also be provided as supplementary material.

Thanks for this suggestion. We have now reported both the non-target probability and random guess probability (Extended Data Fig. 1-1).

Reviewer #2 - Advances the Field

If actually proven effective, the authors demonstrate an interesting and novel approach to subtly manipulate the performance of individual items in multi-item WM tasks.

Reviewer #2 - Statistics

The authors do not correct for multiple comparisons: For example, the rANOVA main effect in question (containing three levels) is not significant for Experiment 3 and the follow-up pairwise comparisons are not corrected, yet the authors conclude that there is in an effect.

Reviewer #2 - Comments to the Authors

The authors demonstrate a novel and interesting approach to manipulate the performance in a multi-item WM task. I have a few concerns about the methodology, statistical analyses, and the conclusions drawn, however.

Major:

1. The authors use target probability from the mixture model as the dependent measure for all main analyses, instead of absolute error or another parameter from the model (i.e. SD). They justify doing so by giving references of other works (line 205). However, apart from their own previous work, the given references actually reported all parameters, as well as raw performance. Indeed, this is also what the authors should do here. In fact, it seems that the number of trials is insufficient to properly model the response distributions, as can be seen in the normalized target probability plots where the performance of many subjects seems to be at the peak of the distribution (at around 5), suggesting that the mixture model estimated a guess-rate of exactly 0 for those subjects. I thus strongly suggest not to use the model on this data, or, at the very least acknowledge the shortcomings and explicitly report the same analyses on the other parameters (most notably SD) and raw performance (this should be moved from the supplemental to the main text and figures).

We thank the reviewer for the important suggestions.

First of all, sorry for the unclear statement. Indeed, the precision results in Figure S2 (renamed as Extended data Fig. 1-2) are estimated by calculating the reciprocal of circular standard deviation of response error (circular difference between reported orientation and correct orientation) across trials (1∕σ).

Second, note that the original study (Bays et al., 2009) used around 50 trials per condition to fit the model. Here we almost doubled the trial number in Experiment 1&2, and used similar trial number in Experiment 3&4. Therefore, the high target probability and the near-zero guess rate suggest that the task is quite easy.

Finally, the target probability in Bays’s study is also close to 1 (the normalized target probability is around 5), while the guess rate is near 0 for a two-item memory task. This probably explains why the enhancement effect is very small especially in Experiment 2. Following reviewer’ suggestion, we have also reported the raw performance in main text and figures. In addition, we have added the non-target probability and guess rate results (Extended Data Fig. 1-1).

2. The authors should acknowledge that many of the results are weak/absent and not statistically significant. Experiment 2 shows a non-significant trend of an effect on the normalized target probability. Figure S2B, suggests that when actually looking at the raw performance, the effect, if anything, might actually be the other way round. I wonder what the SD parameter looks like here. Furthermore, the paired t-tests in Experiments 3&4 should be corrected for multiple comparisons. When done, then only two out of the four experiments have statistically significant effects. I understand that the significance threshold is arbitrary. I suggest complementing all frequentist statistics with Bayesian statistics.

Thanks for raising this concern.

First, as mentioned above, Figure S2 (renamed as Extended data Fig. 1-2) actually plots the memory precision, i.e., the reciprocal of the circular standard deviation of response error. In fact, we do agree with the reviewer that the effect was weak in Experiment 2&4, and hope that the raw performance results for Experiment 2&4 to some extent support the trend.

Following the reviewer’s suggestion, paired t-tests were corrected for multiple comparisons and have been added to Results (Experiment 3: post-hoc analysis: Leader vs. Follower1st: t(29) = 1.88, p = 0.20, Cohen’s d = 0.45; Leader vs. Follower2nd: t(29) = 2.29, p = 0.08, Cohen’s d = 0.55; Follower1st vs. Follower2nd: t(29) = 0.41, p = 0.69, Cohen’s d = 0.10); Experiment 4: post-hoc analysis, Leader vs. Follower1st: t(29) = 2.06, p = 0.09, Cohen’s d = 0.49; Leader vs. Follower2nd: t(29) = 3.57, p = 0.002, Cohen’s d = 0.85; Follower1st vs. Follower2nd: t(29) = 1.51, p = 0.14, Cohen’s d = 0.36).

Furthermore, to verify the consistent findings between experiments, we performed two-sample one-way repeated ANOVA (Experiment (Experiment 1 vs. Experiment 2) * perturbation condition (Leader vs. Follower) based on the normalized target probability. The significant effect of perturbation condition and nonsignificant interaction suggests the consistent memory modulation effect across Experiment 1&2 (experiment * perturbation modulation: F(1,58) = 1.52, p = 0.22, ηp2 = 0.03; perturbation modulation: F(1,58) = 11.29, p = 0.001, ηp2 = 0.16). We also performed two-sample one-way repeated ANOVA for Experiment 3&4, and observed similar results pattern (Experiment * perturbation modulation: F(2,58) = 0.73, p = 0.49, ηp2 = 0.01; perturbation modulation: F(2,58) = 9.11, p < 0.001, ηp2 = 0.14).

Moreover, as suggested by the reviewer, we have also implemented the Bayesian statistic test, which confirms the significant memory modulation effect. Specifically, BF10 is 20.88 for Experiment 1&2, and 180.6 for Experiment 3&4, indicating that the observed data supports the hypothesis for perturbation effect vs. null hypothesis.

Taken together, we thank the reviewer for pointing out the weak statistical effects and for providing the suggestions. We have explicitly stated the weakness in the paper. Hopefully, the added new statistical test results strengthen our conclusion that the dynamic perturbation is consistent across experiments and parameters.

Minor:

1. What does B and C stand for in the bar plots?

Sorry for the confusion. B stands for Leader and C indexes Follower. Corrected.

2. Was the mixture modelling done separately within each sub-condition (i.e. upper and leader, lower and leader, and so on?) How many trials were used for each model?

We pooled sub-condition data before doing mixture modeling. In fact, we have separately applied the mixture modeling for each sub-condition (Extended data Fig. 1-3), which showed a similar but weaker pattern. This might be due to the small number of trials for each sub-condition. Given the nonsignificant effects for both location and interaction, we pooled trials between sub-conditions in the following experiments.

3. Line 207: The authors state that “p” is target probability in the formula. Is that really so? Isn’t it the inverse, that is, guess rate?

The target probability is not exactly the reverse of the guess rate. Instead, in the probabilistic mixture model (Bays et al., 2009), the errors in the reproduction task arise from three sources: (1) the gaussian variability in memory for the target orientation; (2) a certain probability on each trial of misreporting one of the other nontarget orientations; (3) a certain probability of responding with a random orientation not related to any of the items. Therefore, the summation of the probability of reporting the correct target item (target probability), the probability of mistakenly reporting a nontarget item, and the probability of responding randomly would be 1.

4. In Figure S1B a control Experiment is reported, which is not further acknowledged in the main text. Also, what was the a priori reason to suspect that 500 ms would be too long of a time-lag to be effective?

The control experiment using a 500 ms time lag is based on the following reasons. Firstly, previous neurophysiological studies reveal the crucial role of theta band (3-8 Hz) rhythm in memory reactivation and replay (Lisman and Idiart, 1995; Buszaki, 2002). Secondly, our previous study (Li et al., 2021) suggests that the 500 ms temporal lag is out of the short-term plasticity window and couldn’t effectively modulate memory performance. Therefore, here we aimed to test the finding in our current design. To avoid the confusion and make the paper concise, we have removed this part now.

5. In lines 423 to 425 the authors state that the manipulation was unconscious and that subjects could not discriminate the sequences. Was this actually tested?

Sorry that we did not explicitly test the perception here. After participants accomplished the task, we just presented the luminance sequence again and asked them to discriminate. They reported that the two sequences were independent and barely noticed their temporal relationship.
