# Supplementary figures and images for: “Leader–Follower” Dynamic Perturbation Manipulates Multi-Item Working Memory in Humans
Source: eNeuro. 2023 Nov 20;10(11):ENEURO.0472-22.2023. doi: 10.1523/ENEURO.0472-22.2023 (PMC10668215; doi:10.1523/ENEURO.0472-22.2023)

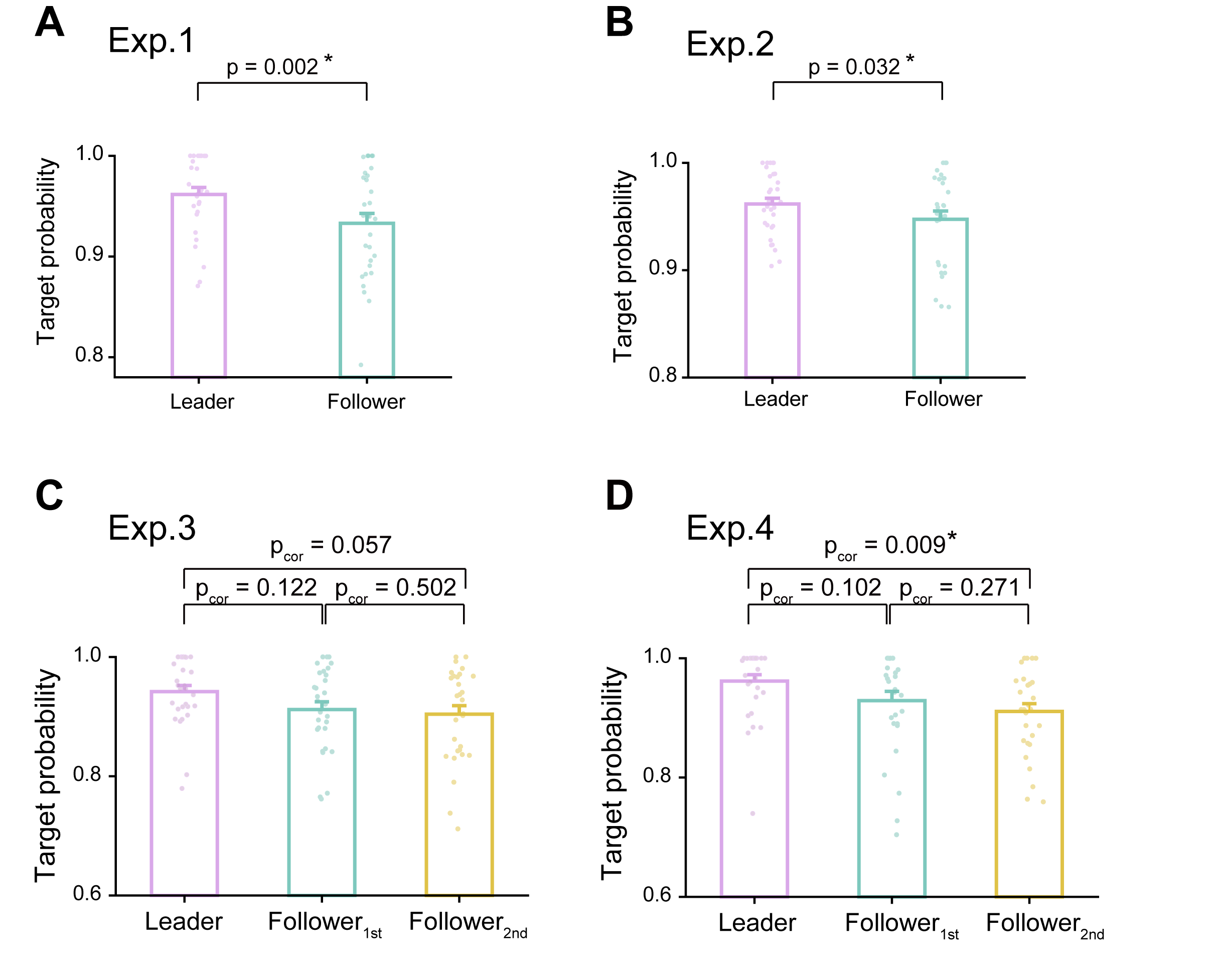

Supplement: Figure 1-1 — A, Target probability for the Leader (purple) and Follower (turquoise) conditions, with dots denoting individual subjects in experiment 1. B–D, Same as A, but for experiments 2–4. Correction for multiple comparisons was applied to experiments 3 and 4. Download Figure 1-1, TIF file. [file enu-eN-NWR-0472-22-s01.tif]

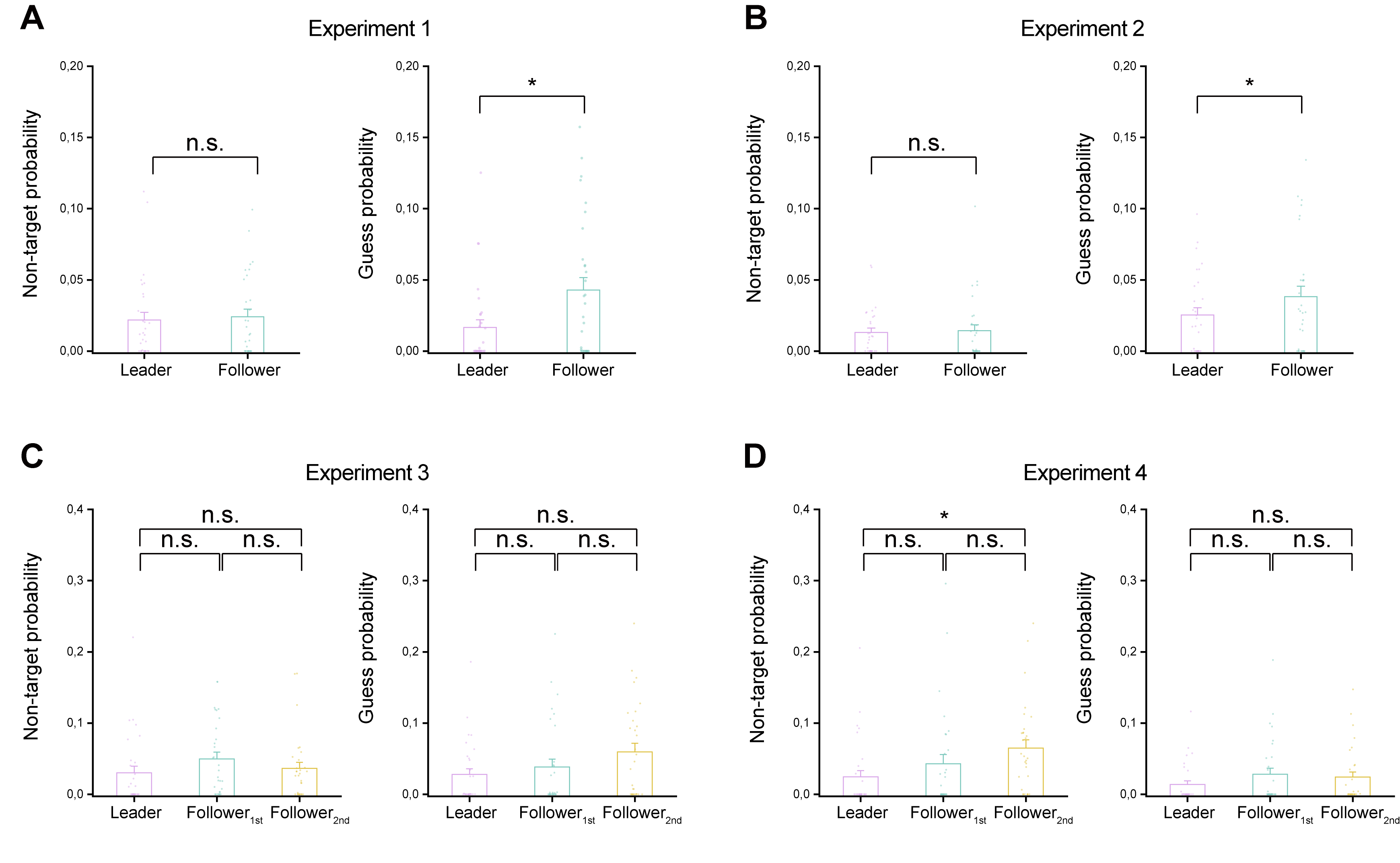

Supplement: Figure 1-2 — A, Left, Nontarget probability for the Leader (purple) and Follower (turquoise) conditions, with dots denoting individual subjects in experiment 1. Right, Random guess probability in experiment 1. B–D, Same as A, but for experiments 2–4. Correction for multiple comparisons was applied to experiments 3 and 4. Download Figure 1-2, TIF file. [file enu-eN-NWR-0472-22-s02.tif]
